# A Two-Year Comparative Evaluation of Clinical Performance of a Nanohybrid Composite Resin to a Flowable Composite Resin

**DOI:** 10.3390/jfb12030051

**Published:** 2021-09-09

**Authors:** Carelle Badr, Gianrico Spagnuolo, Francesco Amenta, Carlos Khairallah, Syed Sarosh Mahdi, Elie Daher, Gopi Battineni, Nadim Z. Baba, Tatiana Zogheib, Syed Saad B. Qasim, Tony Daher, Nalini Chintalapudi, Carina Mehanna Zogheib

**Affiliations:** 1 Department of Esthetic and Restorative Dentistry, School of Dentistry, Saint Joseph University of Beirut, Beirut 1004 2020, Lebanon; carelle.badr@hotmail.com (C.B.); carloskhai@hotmail.com (C.K.); elyedaher@hotmail.com (E.D.); tonydaher@hotmail.com (T.D.); carinamhanna@hotmail.com (C.M.Z.); 2 Department of Neurosciences, Reproductive and Odontostomatological Sciences, University of Naples “Federico II”, 80131 Napoli, Italy; 3 Institute of Dentistry, I. M. Sechenov First Moscow State Medical University, 119435 Moscow, Russia; 4 Center of Clinical Research, Telemedicine & Telepharmacy Department, School of Medicinal and Health Products Sciences, University of Camerino, 62032 Camerino, Italy; francesco.amenta@unicam.it (F.A.); syedsarosh.mahdi@unicam.it (S.S.M.); gopi.battineni@unicam.it (G.B.); nalini.chintalapudi@unicam.it (N.C.); 5 Department of Community Dentistry, Jinnah Medical and Dental College, Sohail University, Karachi 74800, Pakistan; 6 Advanced Specialty Education Program in Implant Dentistry, School of Dentistry, Loma Linda University, Loma Linda, CA 92354, USA; nbaba@llu.edu; 7 Department of Orthodontics and Dentofacial Orthopedics, International University of Catalunya, 08017 Sant Cugat del Vallés, Barcelona, Spain; tatianazogheib@hotmail.com; 8 Faculty of Dentistry, University of Kuwait, Kuwait City 12037, Kuwait; sayed.binqasim@ku.edu.kw

**Keywords:** nanohybrid composite resin, flowable composite resin, class I, class II, clinical study, composite resin

## Abstract

Objective: This prospective in vivo study aimed to compare the clinical behavior of a flowable composite resin (Genial Universal Flo, GC) and a nanohybrid universal composite resin (Tetric Evo Ceram, Ivoclar Vivadent) used in Class I and II direct esthetic restorations in posterior teeth. Methods: A total of 108 Class I and II direct restorations were performed in patients aged between 20 and 60 years. The originality of this study lies in the fact that both materials were placed in pairs, in the same clinical environment (i.e., the same patient and the same type of tooth). The evaluations were performed now of restoration and after 2-weeks, 6-, 12-, and 24-months intervals using clinical examination, clinical photographs, and radiological examination, according to modified USPHS criteria. Statistical analysis was performed using the Fisher exact test and chi-square analysis. Results: At baseline, the universal composite resin showed better esthetic properties such as surface luster, surface staining marginal staining. Both materials regressed significantly over time with no significant difference between groups. Conclusions: Both flowable and nanohybrid composite resins exhibit acceptable clinical performance. The present 24 months of evaluation of different composites showed that the G-ænial Universal Flo could be an effective esthetic material for posterior restoration. No significant difference between both materials over time concerning surface luster, surface staining, and marginal staining.

## 1. Introduction

Composite resins have evolved considerably. Direct composite resin restorations have become an essential part of conservative treatments in restorative dentistry [1]. Their use for the direct restoration of posterior lesions is increasing in dental practice, especially because of the esthetic outcome and noninvasive conservative approach [2]. Multiple-face restorations on permanent premolars and molars are the most frequent types of restorations due to the localization of caries, which first occurs at the occlusal surfaces and subsequently at the proximal surfaces of teeth [3]. 

Several clinical studies have confirmed the use of composite resins to restore Class I and II cavities yielding acceptable and sustainable clinical outcomes [2,4]. Nanohybrid resin composites present conventional particles mixed with nanometric fillers, and therefore, have similar performance to nano filled or micro-hybrid resins. The introduction of well-dispersed inorganic particles into a resin matrix is extremely effective for improving the performance of polymeric composite material. Those composites were introduced to provide a material with high initial polishing combined with superior polish and gloss retention as well as wear resistance [5,6,7]. 

After using the conventional composite for a prolonged period, flowable composite has been introduced to the market, with higher filler content with superior mechanical properties [8]. The use of flowable composite resins has increased dramatically since their introduction in 1995; their main indication was as a base or liner under posterior composite resin restorations. Their fluidity ensures a perfect adaptation to the walls of the cavity, which reduces the risk of air trapping and the formation of voids; this helps to reduce stress at the margin of the restoration [9,10,11,12,13,14,15]. Their composition consists of a reduction in the number of fillers from 20% to 25% and an increase in the monomeric diluents, which results in a decrease in viscosity and an improvement in fluidity. This leads to a decrease in the mechanical and physical properties by increasing the matrix proportion and reducing the quantity of the fillers. To sum up, the flowable composite was not used as a stand-alone restorative option for its increased polymerization shrinkage, reduced mechanical properties, and esthetics [16,17].

A new generation of flowable composite resins can provide improved physical and mechanical properties over conventional composite resins. GC introduced G-ænial Universal Flo (GC America, Alsip, IL, USA), which is a light-cured composite resin claiming to have improved qualities that allow restoring different types of cavities while offering good viscosity and ease of handling. The quantity and the dispersion of the fillers in it may have considerable performances from the mechanical and esthetic point of view; adopting ultrafine strontium glass fillers that provide a reduced risk of filler drop out during occlusal loading due to the small filler size (200 mm) and higher filler load of 69% compared to 20–25% in conventional flowable composite enable the material to achieve high strength and wear resistance [18,19]. Therefore, the study aims to evaluate flowable composite resin against regular composites, because of its easy handling, time-saving, and the gain of extra mechanical properties at the same time representing the importance of the study. The null hypothesis offers no difference between the clinical behavior of the G-ænial Universal Flo flowable composite resin and Tetric Evo Ceram nanohybrid universal flow used in restoring Class I and II direct esthetic restorations on posterior teeth. Tetric Evo Ceram is the nanohybrid universal resin composite and G-ænial Universal Flo is a flowable universal composite. Both are not bulk fill resin composite; they are placed using the incremental technique respectively.

## 2. Materials and Methods

### 2.1. Study Design

A split-mouth design was used. Premolars or molars received the same type of restoration. For risk bias reduction, the selected teeth have been compared with their contralateral ones. Both groups with their corresponding cavities were filled respectively with G-ænial Universal Flo and Tetric Evo Ceram. Table 1 presents the composition details and a comparison of both composite and bonding products used in this study. Both bonding were “one-step, self-etch” applied with selective enamel etching. AdheSE One F respectively for Tetric Evo Ceram and G-ænial bond for G-ænial Universal Flo.

The originality of this study lies in the fact that both materials were placed in pair, in the identical clinical environment (i.e., the same patient and the similar tooth). Randomization was based on the use of the flip of a coin for the choice of the composite resin material. To achieve the balance of randomization into groups, the resin composite that filled the cavity was chosen randomly [20]. The indication for treatment was the replacement of old fillings, deficient restorations, or treatment of primary caries. In the same month, two cavities on two different teeth were prepared. Each one was filled with one of the materials. Informed consent was provided by all participants to follow the periodic reassessment form. The ethical committee of the Saint Joseph University of Beirut has approved this study. 

### 2.2. Inclusion and Exclusion Criteria

We tried to collect the maximum number of patients for more reliability. Assessment started with 56 patients but two were did not came back for clinical assessment. Therefore, a total of 54 patients of which 39 were female and 15 males between 20 and 60 years (Age mean: 40.3 years) have participated. One hundred and eight class I and II direct restorations were received including 62 class I and 46 class II restorations. Participants recruited were referred to the Department of Restorative and Esthetic Dentistry at the Faculty of Dental Medicine, Saint Joseph University of Lebanon. The inclusion criteria employed were:

- Age between 20 and 60 years.
- Good dental hygiene.
- Capacity to read and sign the informed consent form.
- Need for replacement of old fillings and deficient restorations or treatment of primary caries (replacement of defective restoration regardless of the depth of the cavity: The flowable composite resin (G-ænial Universal Flo, GC) and the nanohybrid universal composite resin (Tetric Evo Ceram, Ivoclar Vivadent) used in Class I and II direct esthetic restorations in posterior teeth were used to fill each cavity in incremental horizontal 2-mm layers, polymerized with a light-curing unit (Litex 695, Dentamerica, Industry, CA, USA), at a distance of 2 mm for 20 s on a soft start mode to minimize polymerization shrinkage of composite: curing begins at low light intensity followed by full light intensity to permit grater flow and stress release in the composite. A minimum of 800 mW/cm^2^ of light intensity is provided.
- Confirmed follow-up examinations.

Patients with advanced malocclusion, bruxism, periodontitis, or dentures were excluded from this study.

### 2.3. Application of Resin Fillings 

The placement of restoration was performed by a single trained faculty restorative specialist. Two cavities from the same mouth on two different teeth were prepared and filled with one of the tested materials. The cavities were prepared according to simple, basic geometry: box-shaped (2-mm depth), with a Cavo-surface angle and rounded corners ending in a butt-joint and parallel walls to avoid any ledges. The originality of this study lies in the fact that both materials were placed in pair, in the identical clinical environment (i.e., the same patient and the similar tooth), which means for example: A Class I was performed on tooth #45 and tooth #35. For Class II restorations, a probe was used to determine the depth of the cavities. Every extant lesion was excluded from the study.

Rounded and pear-shaped diamond burs (Intensiv SA, Montagnola, CH, USA) were used for cavity preparation. After rubber dam (Crosstex, Santa Fe Springs, CA, USA) placement, both cavities were bonded: one cavity with G-ænial Bond (GC America, GC America, Alsip, IL, USA) and restored with G-ænial Universal Flo. The other one with AdheSE One F (Ivoclar Vivadent, Amherst, NY, USA) and restored with Tetric Evo Ceram (Ivoclar Vivadent, Amherst, NY, USA) according to the manufacturer’s recommendations. A metallic matrix band was placed with a wooden wedge using Palo dent System (DenstplySirona, York, PA, USA) and then each cavity was filled using the restorative material in incremental horizontal 2-mm layers, polymerized with a light-curing unit (Litex 695, Dentamerica, Industry, CA, USA), at a distance of 2 mm for 20 s. Light curing was started at low light intensity followed by full light intensity to permit greater flow and stress release in the composite. A minimum of 800 mW/cm^2^ of light intensity was provided. The finishing and polishing of the restoration were done with adapted red- and yellow-coded polishing burs (Intensiv) followed by silicone points (Enhance, DenstplySirona, York, PA, USA) and polish paste (DiaPolisher Paste, GC America, Alsip, IL, USA). Bitewing radiographs and clinical pictures were taken with a professional camera (Canon EOS 550D/Canon 100 mm macro lens) after finishing the filling to compare them with those taken in subsequent evaluations. All the pictures were taken in the same room, at the same timing of the day, and using the same settings and specifications of the camera.

### 2.4. Evaluation Criteria 

**Figure 1:**
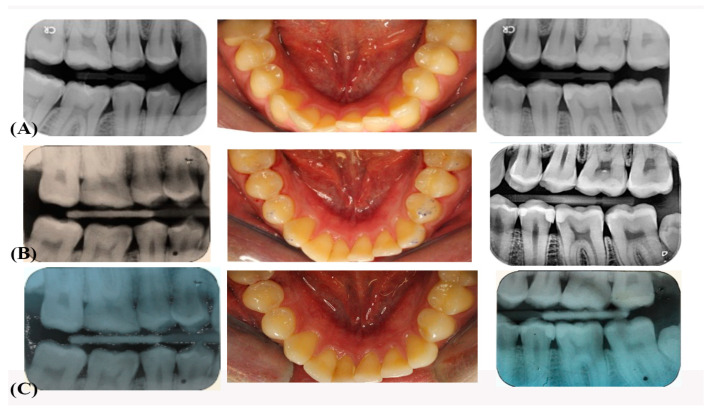
(**A**) Pre-operative clinical photograph and bitewings radiographs on teeth 45 and 35 occluso distally (**B**) Clinical photograph and bitewings radiographs on teeth 45 and 35 occluso distally at T0 (**C**) at 2 weeks.

**Figure 2:**
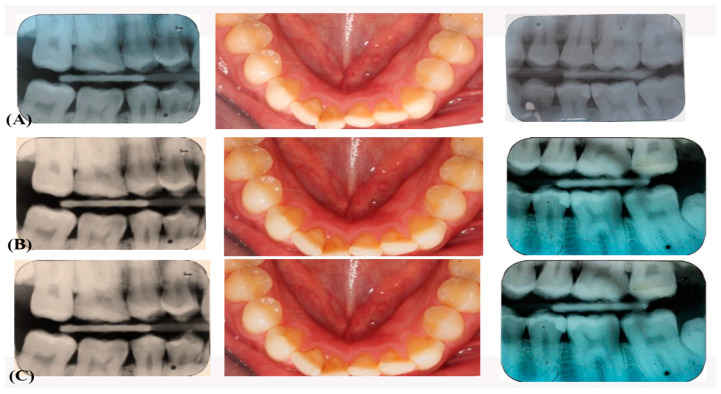
(**A**) Pre-operative Clinical photograph and bitewings radiographs on teeth 45 and 35 occluso distally at 6 months (**B**) Clinical photograph and bitewings radiographs on teeth 45 and 35 occluso distally at 12 (**C**) at 24 months.

The evaluation was performed by two calibrated observers who were blinded to the objective of this study. Both observers did independent evolution of clinical (without clinical pictures) restorations. These evaluations were performed at baseline of 2-weeks, 6-, 12-, and 24-months intervals through a clinical examination, clinical photographs, and radiological examination (see Figure 1 and Figure 2), according to modified United States Public Health Service (USPHS) criteria [21]. Each outcome variable was recorded double by two calibrated investigators. When the two investigators differed by 2% in scoring the restorations, agreements were achieved following detailed discussion. Table 2 presents both esthetic and biologic evolution criteria performed during the tests.

### 2.5. Data Analysis 

Statistical analysis of the data was performed by IBM SPSS v.17. Indicators have been measured based on esthetic and biological criteria. Five indicators were defined as clinically excellent, clinically good, clinically satisfactory, clinically unsatisfactory, and clinically poor (Table 1). Each of these indicators has been studied as a function of two factors such as time and filling materials (i.e., G-ænial Universal Flo and Tetric Evo Ceram). The Fisher and Chi-square (χ^2^) tests were performed to compare scores with time and filling material groups, and the *p*-value (≤0.05) was considered statistically significant.

## 3. Results

### 3.1. Esthetic Criteria

**Figure 3:**
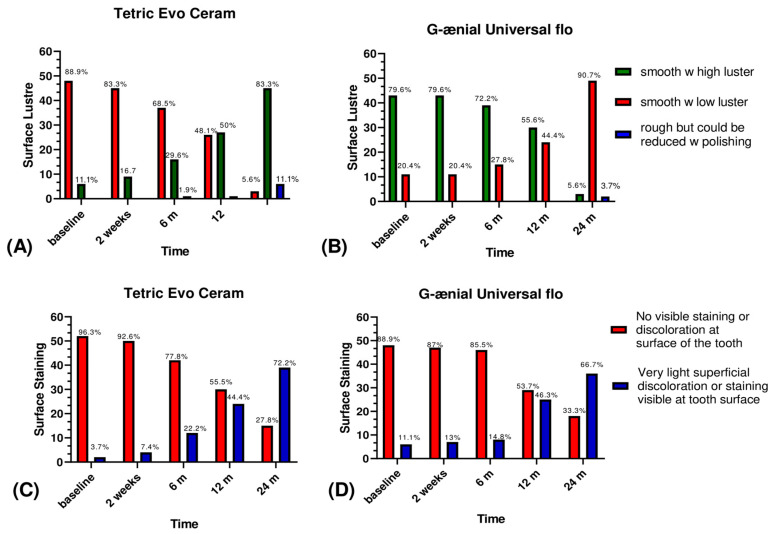
Graphical representation of surface luster and surface staining scores between groups.

In surface luster, 11.1% of the Tetric Evo Ceram restorations and 20.4% of the G-ænial Universal Flo restorations had a low polish area at Baseline (*p* = 0.186). After 24 months, these percentages increased to 83.3% for Tetric Evo Ceram (*p* < 0.001), and to 90.7% for G-ænial Universal Flo (*p* = 0.037) with no significant difference between both resin composites (*p* = 0.420) (Figure 3).

**Figure 4:**
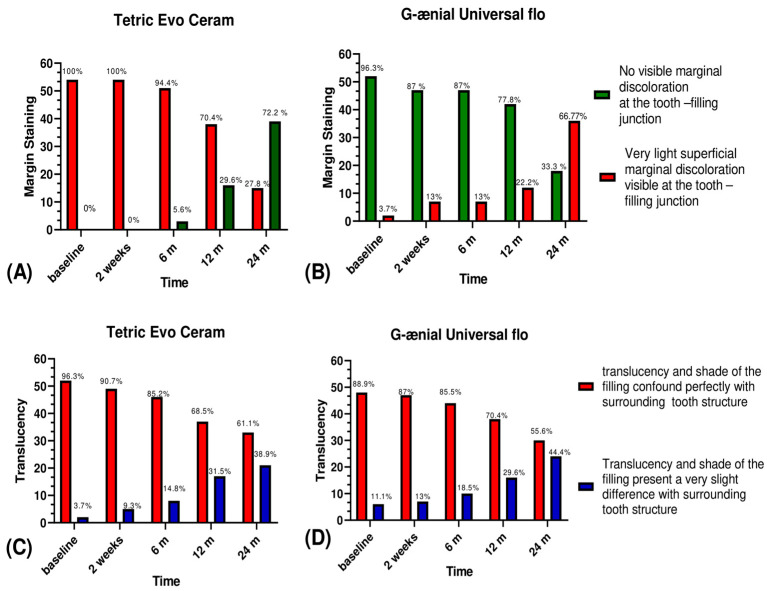
Graphical representation of scores of marginal staining and translucency between groups.

**Figure 5:**
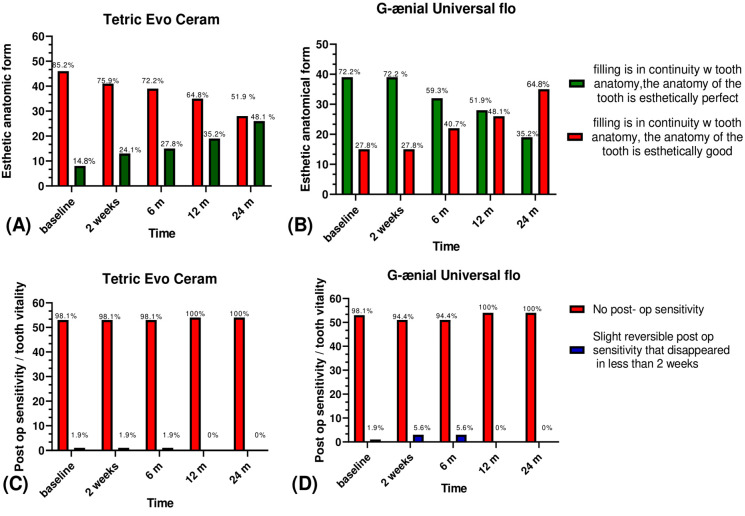
Graphical representation of esthetic anatomic form and post op sensitivity/tooth vitality scores between groups.

In surface staining, 3.7% of Tetric Evo Ceram and 11.1% of G-ænial Universal Flo restorations had a very slight superficial discoloration at baseline (*p* = 0.291). After 24 months, these percentages increased to (72.2% for Tetric Evo Ceram (*p* < 0.001), and to 66.7% for G-ænial Universal Flow (*p* < 0.001); with no significant difference found between both materials (*p* > 0.05) (Figure 3). Moreover, no Tetric Evo Ceram and 3.7% of G-ænial Universal Flo restorations had visible marginal discoloration at the tooth-restoration joint at baseline (*p* = 0.153). After 24 months, these percentages increased to 51.9% for Tetric Evo Ceram (*p* < 0.001), and 42.6% for G-ænial Universal Flo (*p* < 0.001) with no significant difference found between both materials (*p* = 0.335). Within the translucency criteria, 3.7% of Tetric Evo Ceram restorations and 11.1% of G-ænial Universal Flo restorations showed a very slightly different color with the surrounding tooth at baseline (*p* = 0.270). At 24 months, these percentages increased significantly to 38.9% for Tetric Evo Ceram (*p* < 0.001) and 44.4% for G-ænial Universal Flo (*p* < 0.001). Translucency was not significantly different between Tetric Evo Ceram and G-ænial Universal Flo after 24 months (*p* > 0.05) (Figure 4). Finally, in esthetic anatomical form, Tetric Evo Ceram showed 14.8% of restorations were aesthetically good at baseline and this percentage increased significantly to 48,1% after 24 months (*p* < 0.001). With G-ænial Universal Flo, 27.8% of restorations were aesthetically good at baseline and this percentage increased significantly to 64.8% after two years (*p* < 0.001). The anatomical shape was significantly different between both materials after 24 months (*p* = 0.034) (Figure 5).

### 3.2. Biological Criteria

At baseline, 1.9% of teeth treated with Tetric Evo Ceram and 1.9% of teeth treated with G-ænial Universal Flo showed a slight reversible postoperative sensitivity. This percentage did not significantly change over time for Tetric Evo Ceram (*p* = 1.000) and G-ænial Universal Flo (*p* = 0.376). Our study revealed that all the restorations with both materials showed no caries at the tooth-filling junction over time. In periodontal response, no inflammation around any restoration was noted at baseline for both groups. The percentage did not significantly change over time for both materials: Tetric Evo Ceram (*p* > 0.05) and G-ænial Universal Flo (*p* > 0.05) (Figure 5).

## 4. Discussion

Nowadays, short-term studies have a big impact on the early prediction of clinical performance and longevity of restorations [22,23,24]. Despite all the variables, the only clinical environment can provide a reliable evaluation of dental materials and restorative techniques [25]. Clinical trials require reliable objectives and criteria to evaluate the performance of restorations. Generally, a well-designed prospective clinical study is still better than a retrospective study that provides data that were collected for other purposes. In this study, two posterior composite resins (one flowable and one nanohybrid universal) were evaluated in vivo for clinical performance over 24 months based on USPHS criteria for Class I and II cavities in permanent premolars and molars. Follow-up visits during this study were performed on 100% of the restorations.

At baseline, the nanohybrid material showed a better surface luster than the flowable composite resin with *p* < *0*.05. After 24 months, the surface luster of these two materials significantly regressed to 50%, with *p* < 0.001 for Tetric Evo Ceram and *p* = *0*.037 for G-ænial Universal Flo, with no significant difference between both materials (*p* = *0*.420). Concerning the surface staining, at baseline, the universal nanohybrid composite resin showed better surface condition than the flowable composite resin. After 24 months, both materials revealed significant superficial discoloration (*p* < 0.001) for Tetric Evo Ceram and *p* < 0.001 for G-ænial Universal Flo, with no significant difference between both materials (*p* > 0.05). Nanohybrid composite resins such as Tetric Evo Ceram contain 500-nm medium-sized barium glass mineral fillers, with better polish capacities and a higher surface state than the flowable composite resin at the baseline. However, this was not directly constant over time. In contrast with the Tetric Evo Ceram, the G-ænial Universal Flo, also contains mineral fillers such as silicon dioxide, depicting a constant behavior without regression [26,27].

The most significant changes in commercial composite resins in recent years have been the modification in the content of mineral fillers. The size of the fillers incorporated in the matrix of new composite resins has been reduced, leading to nano filled composite resins with improved properties. Several studies have reported that nanocomposite resins (nanohybrids or nano filled) have a better surface polish and lower abrasion resistance. Mitra et al. found that nano filled composite resins wear out by detaching particles or individual parts of nanoclusters, rather than by detaching larger particles, resulting in a relatively smooth wear surface [28]. Nanofluids such as G-ænial Universal Flo exhibit an improvement in polymerization shrinkage because it is a resin system that is claimed to control polymerization kinetics having incorporated nanosized fillers, and an improvement in surface finish, polishing, and mechanical properties [14].

G-ænial Universal Flo can achieve physical performance comparable to or better than conventional composites especially with regard to the high flexural strength and wear resistance, due to its homogeneously dispersed and extremely fine silane treated filler particles [29]. These fillers also make it possible to obtain a high gloss in very few steps and to increase the gloss of unpolished surfaces over time (due to its self-polishing property). That is why the material can be used as stand-alone restorative material.

On the other hand, The Tetric EvoCeram composite is a light-curing, radiopaque nanohybrid composite that is based on the latest technology for direct restorative therapy. It is new universal material from Vivadent Ivoclar, which is utilized as anterior restorations, class V restorations, veneering of discolored anterior teeth, splinting of mobile teeth, and extended fissure sealing in molars and premolars. Nanoparticles present improved mechanical properties of the material and enable the dentist to choose a better filling color. Properties of the material are similar to hybrid and micro filler composites properties.

Regarding marginal staining, there was no significant difference between the two groups at baseline (*p* = 0.153); no visible marginal discoloration was found at the tooth-restoration joint for Tetric Evo Ceram compared to G-ænial Universal Flo restorations. There was a 3.7% of slight superficial marginal discolorations. There was a statistical increase for both groups over two years (*p* < 0.001) for Tetric Evo Ceram and <0.001 for G-ænial Universal Flo with no significant difference found between both materials (*p* = 0.335). These results are in line with the study of Gallo et al 2006, which clinically evaluated two-fluid composite resins: Tetric Flow (Ivoclar Vivadent) and Esthet-X Flow (Dentsply Sirona) over one year [30]. Marginal discoloration statistically increased from baseline to one year [31,32,33].

Concerning the translucency, Tetric Evo Ceram was better, but over time a slight difference with the surrounding tooth structure was noted for both materials. On the other hand, translucency was not significantly different between the two groups with *p* > 0.05. G-ænial Universal Flo offers a choice of 15 different shades to suit different clinical situations. According to the manufacturer, the optical properties of G-ænial Universal Flo are superior to flowable composite resin and be even superior to the properties of tested nanohybrid composite resins with a choice of 19 shades. Over time, there was a very significant difference between G-ænial Universal Flo and Tetric Evo Ceram [34]. From an esthetic anatomical point of view, at baseline, the Tetric Evo Ceram restorations were more esthetic than the G-ænial Universal Flo restorations. This may have been due to the consistency of the fluid flowable, which makes the reproduction of the anatomy and the sculpture of the restoration more delicate, even if the handling of G-ænial Universal Flo is easier than other conventional flowable, as it is more viscous. The viscosity of G-ænial Universal Flo is indeed higher than other fluid composite resins and has been designed to improve the handling of the material while restoring cavities. It has thixotropic properties, which means it stays in place to allow for the sculpting of the material after its placement using a probe, for example.

Over time, these two materials lost significant esthetics. At baseline, Tetric Evo Ceram was esthetically better. At 24 months, no significant difference regarding esthetics was noted between the two materials. Recently, composite resins containing nanometric fillers have progressed by improving their surface condition, polishing ability, and mechanical properties [29,35]. Flowable composite resins have also been improved by incorporating nanosized fillers into their composition to restore the occlusal cavities of posterior teeth [33,34,35,36,37]. 

All participants were satisfied with their restorations over time. Besides, a complete absence of secondary caries in both groups was observed because all the patients were maintaining good oral hygiene. This was verified by the bitewing radiographs taken at each control visit. In 2012, Demarco et al. reported that the development of secondary caries is due not only to the material but also to the clinical environment, the patient’s hygiene, and the different handling techniques of the material. All of these factors could also affect clinical outcomes [38,39,40,41,42].

Regarding postoperative sensitivity, there was no statistically significant difference between Tetric Evo Ceram and G-ænial Universal Flo. It should be mentioned that there were two cases of postoperative sensitivity, one with G-ænial Universal Flo and the other with Tetric Evo Ceram. Indeed, the major disadvantage of composite resins remains the polymerization shrinkage causing postoperative sensitivity, micro gap formation, and secondary caries [43,44]. In this study, the use of the layering technique and the soft-start polymerization mode may have reduced this shrinkage and thus the postoperative sensitivity. For the periodontal response, no gingival inflammation was observed in either one of the groups. This percentage has not changed significantly over time. There were a few limitations within the study. Firstly, the relatively lowest participant number can hamper the outcome speculations among the population of dental patients. The cavities were not standardized, because the operator was obliged to remove the decay, and this was relative to each patient. The null hypothesis was accepted.

## 5. Conclusions

Within the limitation of this randomized clinical study, the results indicate that the flowable nanohybrid composite showed similar clinical performances in posterior direct restorations in Class I and II cavities in comparison to traditional nanohybrid composite at the end of a 24-month evaluation period. The G-ænial Universal Flo being a new nano filled flowable composite resin, has shown comparable clinical effectiveness as conventional flowable resins; surface luster, surface staining, and marginal staining had no significant difference between both materials over time but concerning translucency a significant difference was noted in favor of Tetric Evo Ceram. These two materials lost significant esthetics.

## Figures and Tables

**Table 1:** Composition details and comparison of both composite and bonding products.

Material	Resin	Filler	Filler wt/vol %	Shade
Tetric Evo Ceram Ivoclar Vivadent, Schaan, Lichtenstein	Bis-GMA, UDMA, Ethoxylated Bis-EMA	Barium Glass, mixed oxide, filler, YbF3, prepolymers	75–76/53–55	
G-ænial Universal Flo, GC corporation, Tokyo, Japan	UDMA, Bis-MEPP, TEGDMA,	Silicon Dioxide, Strontium glass, pigmen	69/50	
**Material**	**Composition**		**Application**	
G-ænial Bond GC, Corporation, Tokyo, Japan	4-META, TEGDMA, UDMA,		Apply 1 coat of Adhesive, leave undisturbed for 10 s, Air dry vigorously, Light cure for 10 s	
AdheSE One Ivoclar Vivadent, Schaan, Lichtenstein	Bis-Acryl, Bis-Meth, H_2_PO_4_, Amino acid Acryl, -OH alkyl Methacryl			

Urethane dimethacylate (UDMA), Methacryloxyethyltrimellitate anhydride (4-META), Triethylene glycol dimethacrylates (TEGDMA), Acrylamide (Acryl).

**Table 2:** Evaluation criteria of esthetic and biological procedures.

			Clinically Excellent/Very Good	Clinically Good	Clinically Sufficient/Satisfactory	Clinically Unsatisfactory	Clinically Poor
Esthetic criteria	Surface luster		The surface of the filling is smooth with high luster	The surface of the filling is smooth but with a low luster	The surface of the filling is rough but could be reduced with a polishing	The surface of the filling is rough but could not be reduced with a polishing	The surface of the filling is severely rough with deep or irregular grooves
	Staining	Surface	No visible staining or discolouration at the surface of the tooth	Very light superficial discolouration or staining is visible at the tooth surface	Visible staining and discolouration at the tooth surface could be removed by polishing	Visible staining and discolouration at the tooth surface with deep penetration that could not be removed by polishing	Very deep surface discolouration
		Margin	No visible marginal discolouration at the tooth-filling junction	Very light superficial marginal discolouration is visible at the tooth-filling junction	Visible marginal discolouration at the tooth-filling junction that could be removed by polishing	Visible marginal discolouration at the tooth-filling junction with deep penetration that could not be removed by polishing	Very deep marginal discolouration
	Colour match and translucency		The translucency and shade of the filling confound perfectly with the surrounding tooth structure	The translucency and shade of the filling present a very slight difference with the surrounding tooth structure	The translucency and shade of the filling do not confound perfectly with the surrounding tooth structure but the difference remains acceptable	The translucency and shade of the filling do not confound perfectly with the surrounding tooth structure. The difference is important	The shade difference is severe
	Esthetic anatomical form		The filling is in continuity with tooth anatomy, the anatomy of the tooth is esthetically perfect	The filling is in continuity with tooth anatomy, the anatomy of the tooth is esthetically good	The anatomy of the tooth is not at all esthetic but still clinically acceptable	The anatomy of the tooth is not at all respected	The anatomy of the tooth is completely lost.
Biological criteria	Postoperative sensitivity and tooth vitality		No post-op sensitivity	Slight reversible post-op sensitivity that disappeared in less than 2 weeks	Slight post-op sensitivity	Severe post-op sensitivity	Severe irreversible post-op sensitivity that led to root canal treatment
	Recurrence of caries, erosion and abfraction		No caries at the tooth-filling junction				Caries detected at the tooth-filling junction
	Tooth integrity		-	-	-	-	-
	Periodontal response		No inflammation. Good oral health	Slight inflammation around the restoration			Severe inflammation around the restoration. Bleeding and pain

## Data Availability

Not applicable.
